# Journal-specific Regulations for Writing and Submitting Papers: Burden for Both Authors and Journals

**DOI:** 10.31662/jmaj.2023-0175

**Published:** 2024-02-05

**Authors:** Shigeki Matsubara

**Affiliations:** 1 Department of Obstetrics and Gynecology, Jichi Medical University, Tochigi, Japan; 2 Department of Obstetrics and Gynecology, Koga Red Cross Hospital, Koga, Japan; 3 Medical Examination Center, Ibaraki Western Medical Center, Chikusei, Japan

**Keywords:** author guidelines, journals, submission, writing papers

## Dear Editors:

Teixeira da Silva and Daly ^(1)^ state that journal editors and authors should be mindful of the International Committee of Medical Journal Editors (ICMJE) recommendations ^(2)^. I agree with it. I wish to add one important point regarding writing format and submission, which was not mentioned ^(1)^. Many journals have writing formats and/or submission guidelines specific to them. Nonadherence prolongs submission processes, annoying both authors and journals.

Over 4 decades, I wrote 296 first-authored PubMed-indexed papers. My main targets were 7 Obstetrics and Gynecology (OBGYN) journals from the international journals in the USA, UK, Germany, France, Scandinavia, and Japan. Besides ICMJE, these journals have specific guidelines. The examples are as following: i) authors’ signatures are, or are not, required, and if required, in a covering letter or in their own format; ii) word-count should be given on the title page or in a covering letter; iii) font should be Times New Roman or Arial, and iv) line numbers should, or should not, be added. Nonadherence leads to manuscript return: several days are required from initial to resubmission. Paper return simply because of the nonuse of Arial font is discouraging. Extra efforts are required for both authors and journals.

I used to be an Editor-in-Chief (EIC) of some journals. I also added some specific guidelines in my EIC era, considering journals’ specificities. Every journal has its own tradition, policy, and style. I would never criticize the required specific guidelines.

It would be author-friendly for the journal side to make the required changes when it comes to simple format modifications. However, my EIC experience made me recognize journals’ limited man-power, and some journals are concerned about making inappropriate changes. Some journals may consider that adherence and making changes are the authors’ responsibilities.

The following may be worth considering. I did not think of it in my EIC era. The problem is: both journal-specific and general ICMJE guidelines are described in Instructions to Authors in a mixed and standard manner. Journals should state “guidelines specific to this journal” at the beginning of the Instructions; for example, “Arial font, signed cover letter with wordcount, and no line numbers,” and “otherwise adhere to the latest ICMJE recommendations (similar to other journals).” This simple note would facilitate paper writing, submission, and editorial processes.

Situations for OBGYN journals may hold true for many Japanese journals. Hopefully, the leadership of JMA Journal will take this issue under consideration.

## Article Information

### Conflicts of Interest

None

### Acknowledgement

I thank Teppei Matsubara (Massachusetts General Hospital, Harvard Medical School, USA) and Daisuke Matsubara (Jichi Medical University, Japan) for their help.

### Author Contributions

S. Matsubara: Identification of the significance and manuscript writing.

### Approval by Institutional Review Board (IRB)

Not applicable

### Data Availability

Data sharing is not applicable to this article, as no new data were created or analyzed in this study.

### Patient Anonymity

Not applicable

### Informed Consent

Not applicable
